# Serum IGF-1 levels as a clinical tool for optimizing orthodontic treatment timing

**DOI:** 10.1186/2196-1042-14-46

**Published:** 2013-11-14

**Authors:** Sapna Jain, Sandhya Jain, Anuradha Deoskar, VS Sai Prasad

**Affiliations:** Department of Orthodontics & Dentofacial Orthopedics, Government College of Dentistry, Opp. Maharaja Yashwantrao Hospital, Indore, India; IARI-regional section, Indore, India

## Abstract

**Background:**

The study aims to associate serum insulin-like growth factor-1 (IGF-1) levels with cervical maturation stages (CS) 3, 4, and 5 on lateral cephalogram in male subjects and to find out peak serum IGF-1 levels among these three stages.

**Methods:**

The study was conducted on 45 male subjects who were at skeletal maturation stage CS-3, CS-4, and CS-5. Subjects were selected using simple random sampling technique. Serum IGF-1 levels were estimated from blood samples using chemiluminescence immunoassay (CLIA) method. CS was evaluated using a six-stage method of evaluating the cervical vertebrae. Mean IGF-1 levels between the stages were compared by analysis of variance (ANOVA) test.

**Results:**

One-way ANOVA showed highly significant differences between all cervical stages with *p* value <0.01, but *post hoc* Tukey test showed highly significant differences between CS-4 and CS-5 with *p* value 0.006. Serum IGF-1 levels showed good association with skeletal age in male subjects; 53.3% of subjects at CS-3, 66.7% subjects at CS-4, and 6.7% subjects at CS-5 showed IGF-1 levels in peak range.

**Conclusions:**

Serum IGF levels can be used as an additional tool to optimize orthodontic treatment timing.

## Background

The assessment of growth potential is essential because of individual variations in timing, duration, and velocity of growth. The classic method of assessing skeletal maturity is by the use of a hand-wrist radiograph. Assessing the degree of cervical vertebral maturation on lateral cephalometric radiograph [1–8] and recording MP3 stages [9, 10] on periapical X-ray films are also used to identify peak mandibular bone growth and skeletal maturity. Radiographic methods, which are highly subjective techniques, involve radiation exposure. More recently, the use of some additional methods with cervical maturation method for the assessment of biologic maturity is proposed [11, 12]. If the shape of the cervical vertebrae does not show the exact stage, then additional method can be of use to predict the maturation stage accurately.

Gingival crevicular fluid alkaline phosphatase level may be used as a noninvasive clinical biomarker for the identification of pubertal growth spurt but only in periodontally healthy subjects [13]. Insulin-like growth factor-1 (IGF-1) is one of the main mediators of the actions of growth hormone in promoting muscular and skeletal growth [14–16]. Parallel to the increase in growth hormone secretion at puberty, circulating IGF-1 also increases [17]. A study has shown that the condyle is more responsive and sensitive to IGF-1 than the femoral head [18]. Mean IGF-1 blood spot levels in the late pubertal stages have been observed to be higher than in the prepubertal, early pubertal, and postpubertal stages [19]. IGF-1 levels were highest at the hand-wrist stages that are previously associated with the greatest amount of mandibular growth [20].

Reference ranges of serum IGF-1 levels in male and female subjects have been established separately according to chronological age [21]. Age-related increase occurs in serum IGF-1 levels during the prepubertal and early pubertal stages and a decrease in late puberty, but chronological age is not a reliable indicator for skeletal maturity [22–24].

Since IGF-1 levels vary according to age and sex [21], separate studies are needed for male and female subjects. The pubertal peak occurs approximately 2 years earlier in girls than in boys [25]. Serum IGF-1 level is a reliable maturation factor for the assessment of skeletal maturity [26]. Since serum IGF-1 quantitatively assesses growth, the intensity of growth can therefore be estimated which would further help in assessing the exact timing of treatment.

The purpose of this study was to associate serum IGF-1 levels with cervical vertebrae maturation stages so as to find out whether IGF-1 levels can be used as a clinical tool or not. The objective was to find out peak serum IGF-1 levels corresponding to cervical vertebrae maturation stages (CS) 3, 4, and 5 in circumpubertal male subjects.

## Methods

The sample used in the study consisted of 45 male subjects, who were at CS-3, CS-4, and CS-5, selected from the patients coming to the Department of Orthodontics and Dentofacial Orthopedics and Department of Pedodontics at our institute by simple random technique. Patients meeting the set selection criteria were selected from the daily patient register until a sample size of 15 was reached in each group to eliminate both deliberate and unconscious bias. Inclusion criteria were male subjects who were at CS-3, CS-4, and CS-5 based on information about birth date and their good health status. Exclusion criteria were subjects suffering from any serious illness; growth abnormality, e.g., craniofacial syndromes, rickets, medical syndromes, bone disease, and bone deformities; bleeding disorders, or history of any serious trauma or injury to the face. The research protocol was approved by the ethical committee of the Government College of Dentistry, Indore, India. Parental/patient's informed consent was taken for enrolment of each subject in the study.

Lateral cephalograms were taken in natural head position within an hour of blood collection. All the radiographs were exposed at 80 kVp, 9 mA for 1.25 s. The six-stage cervical staging technique by Baccetti et al. [6] was used to determine the stages of cervical vertebrae maturation. The observer was blinded about each patient's age and IGF-1 levels. A double determination was carried out on all the 45 individual's radiographs by the same operator at a second session after 15 days. These tracings were analyzed separately, and two sets of readings were obtained for statistical evaluation.

Blood samples were collected between OPD hours of 9:00 a.m. and 12:00 p.m., and serum was separated from the blood samples, labelled with patient's name, properly sealed, stored in a thermocol box with ice pack (kept at 2°C to 8°C), and sent to the laboratory for chemiluminescence immunoassay for determination of IGF-1 levels by a fully automated, two-site chemiluminescent immunoassay (Siemens Immunolite 2000 (Siemens, Munich, Germany) immunoassay machine at Metropolis Laboratories). Each patient's height and weight were measured as they might affect serum IGF-1 levels.

### Statistical analysis

The Pearson correlation was used to measure intra-examiner reliability. The mean Pearson correlation for all variable was 0.932, and it ranged from 0.801 to 0.988, which implies a highly significant correlation between the observations (*p* = <0.01).

Analysis of variance (ANOVA) and *post hoc* tests were used to compare mean IGF-1 levels and cervical vertebral maturation stages. All statistical analyses were performed using SPSS18 for Windows software (version 18.0; SPSS, Chicago, IL, USA). The significance level was set at *p* < 0.01. Student's *t* test was applied to show significant differences between the orthodontic classes (CS-5) and orthopedic groups (CS-3 and CS-4).

## Results

Differences were observed between the cervical stages (*p* < 0.01) when analyzed by one-way ANOVA (Table 1); highly significant differences were found between CS-4 and CS-5 (*p* = 0.006), whereas nonsignificant differences were found between CS-3 and CS-4, and between CS-3 and CS-5 by *post hoc* Tukey test (Table 2).

**Table 1:** **Descriptive IGF-1 (ng/ml) statistics for each cervical stage**

Cervical stage	Subjects	Mean IGF-1 (ng/ml)	SD	95% confidence interval for mean		Minimum	Maximum
				Lower bound	Upper bound		
CS-3	15	318	17.4	283	353	171	433
CS-4	15	352	17.7	317	387	252	525
CS-5	15	279	10.8	257	301	206	372

ANOVA: *p* value = 0.008, highly significant. SD, standard deviation.

**Table 2:** ***Post hoc*** **analysis (least significant difference comparing IGF-1 serum levels (ng/ml)) for CS-3,** CS-**4, and** CS-**5**

Group (mean)	Compared group	Mean difference	SE	***p***value
CS-3 (318.13)	CS-4 (352.40)	−34.27	22.05	0.277
				NS
CS-4 (352.40)	CS-5 (279.67)	72.73	22.05	0.006
				Sig
CS-5 (279.67)	CS-3 (318.13)	−38.47	22.05	0.201
				NS

NS, nonsignificant (*p* > 0.05); Sig, significant (*p* < 0.05); SE, standard error.

Peak IGF-1 level was found in CS-4. In the CS-5 group, IGF-1 level was consistently on the lower hub in all three maturity groups, suggesting the fact that most patients attain their maximum growth by the time of CS-5 (Table 3).

**Table 3:** **Mean serum IGF-1, mean height, and mean weight with respect to various CS in different types of maturers**

Cervical stage	Mean age (years)	95% confidence interval (years)		Advance maturer			Average maturer			Delayed maturer		
		Lower bound	Upper bound	Mean IGF-1 (ng/ml)	Mean height (in.)	Mean weight (kg)	Mean IGF-1 (ng/ml)	Mean height (in.)	Mean weight (kg)	Mean IGF-1 (ng/ml)	Mean height (in.)	Mean weight (kg)
CS-3	13.4	13.2	13.7	317	61.9	40.2	288	61.3	39.8	339	61.6	42.1
				(226–372)	(59–64)	(36–43)	(171–369)	(58–62)	(38–42)	(253–389)	(59–65)	(39–46)
				(<13.2 years)			(13.2–13.7 years)			(>13.7 years)		
CS-4	15.9	15.6	16.2	381	65.2	42.4	318	65.2	42.8	352	66.3	48.2
				(300–525)	(62–68)	(38–46)	(252–398)	(58–71)	(37–52)	(275–427)	(64–68)	(42–50)
				(<15.6 years)			(15.6–16.2 years)			(>16.2 years)		
CS-5	17.3	17.0	17.5	293	67.6	51.1	278	68.0	57.1	267	67.5	52.6
				(222–372)	(66–68)	(45–56.5)	(206–311)	(65–70)	(52–64)	(241–304)	(67–68)	(48–60)
				(<17.0 years)			(17.0–17.5 years)			(>17.5 years)		

Advance maturers, subjects with age less than the lower bound age limit in each cervical stage. Average maturers, subjects with age within the upper and lower bound age limit in each cervical stage. Delayed maturers, subjects with age above the upper bound age limit in each cervical stage.

Table 4 shows the mean IGF-1 level with respect to orthodontic and orthopedic groups. By Student's *t* test, highly significant differences were observed between the classes and serum IGF-1 level with a *p* value of 0.006.

**Table 4:** **Mean IGF-1 level (ng/ml) with respect to orthopedic and orthodontic treatment class**

Group	Subjects	Mean IGF-1 (ng/ml)	SD	Median	95% confidence interval for mean		***p***value
					Lower bound	Upper bound	
Class 1	30	335	12.6	333	310	360	0.006
Class 2	15	280	10.8	285	258	302	

SD, standard deviation; Class 1, orthopedic treatment class; Class 2, orthodontic treatment class.

**Figure 1:**
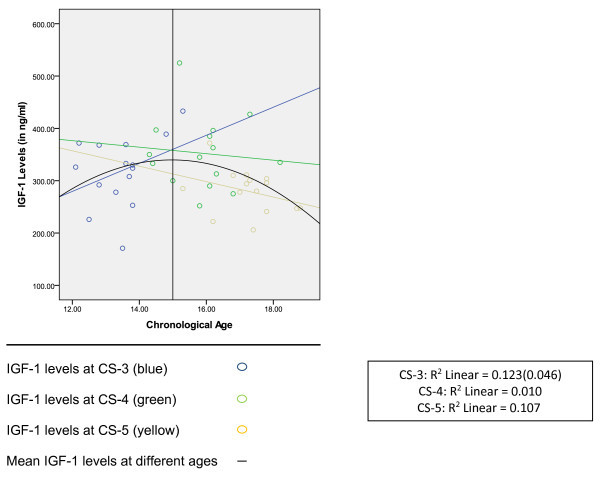
**Scatter curve showing change in IGF-1 levels with age within the same cervical stage.**

The scatter curve (Figure 1) showed that IGF-1 level increased with increasing age at CS-3 and decreased with age at CS-4 and CS-5. Serum IGF-1 level increased up to 15 years of age and decreased thereafter.

## Discussion

The present study was done to evaluate serum IGF-1 levels in CS-3, CS-4, and CS-5 (circumpubertal growth period) because these stages are clinically more relevant stages with the orthodontic speciality. The cervical staging determination method used which was given by Baccetti et al. [6] is considered to be a better method in comparison to other methods [27].

In the present study, mean peak serum IGF-1 level in male subjects at CS-4 (352 ng/ml) was observed, in contrast to a similar study where mean peak IGF-1 level at CS-3 (397 ng/ml) was noticed in female subjects [28]. A possible explanation for the difference observed in stages where peak in IGF-1 levels between the two studies is observed could be related to gender difference since the timing of puberty differs in male and female subjects [1]. This is in accordance with the statement by Proffit [29]: “Girls mature earlier, and finish their growth much sooner. The differences arise because in males slow but steady growth occurs before the growth spurt.” It was also observed that 53.3% (8 out of 15) of the subjects at CS-3, 66.7% of the subjects (10 out of 15) at CS-4, and 6.7% of the subjects (1 out of 15) at CS-5 showed IGF-1 levels in peak range.

The scatter curve showed that serum IGF-1 levels increased with age in CS-3 and decreased with age in CS-4 and CS-5, which means that the peak in serum IGF-1 may occur at the fag end of CS-3 and at the beginning of CS-4. The observed peak IGF-1 levels at CS-3 and CS-4 in the present study coincide with cervical stages with peak mandibular growth, observed by Baccetti et al. [6] in 2005.

The mean serum IGF-1 level in the CS-4 group is 352 ± 17.7 ng/ml with a mean age of 15.9 years, which is in accordance with the study by Brabant et al. [21], in which the highest mean IGF-1 was 382 ng/ml for males which occurred at the age of 15 to 16 years. Studies [21, 25] have attempted to establish a reference range of serum IGF-1 levels according to chronological age in male and female subjects separately, but several studies have shown that chronological age is a poor predictor of the pubertal growth spurt [22–24].

The present study showed the range of serum IGF-1 levels as 171 to 433 ng/ml for CS-3, 252 to 525 ng/ml for CS-4, and 206 to 372 ng/ml for CS-5. The clinical usefulness of the study is limited because of overlapping levels of serum IGF-1 in all three cervical stages, possibly due to the relatively small sample size, cross-sectional design of the study, different body types (aesthetic/athletic/plethoric), and different maturational groups (advanced/average/delayed).

In order to use IGF-1 levels as a diagnostic tool in clinical orthopedic case selection and for establishing a cutoff reference value of IGF-1 for different types of treatments, the subjects in CS-3, CS-4, and CS-5 were divided into two groups. Since *post hoc* analysis revealed no statistical difference in CS-3 and CS-4, therefore, the subjects at CS-3 and CS-4 were combined and considered as class 1 (orthopedic treatment type) in which orthopedic corrections are possible, and subjects at CS-5 were considered as class-2 (orthodontic treatment type). After statistical analysis of these two treatment classes, new upper and lower bound limits of IGF-1 levels were obtained which were 310 and 360 ng/ml, respectively, for orthopedic treatment class and 258 and 302 ng/ml for orthodontic treatment class. Based on these upper and lower bound values, a cutoff value of serum IGF-1 levels for various treatment options was obtained as 310 ng/ml. This means that in patients with serum IGF-1 levels >310 ng/ml, orthopedic treatment may be considered.

To gain additional information about maturational status of growth, the sample was further divided into three groups (advance, average, and delayed maturer) in each cervical stage on the basis of lower and upper bound limits of age. Samples below the lower bound limit of age were considered as advance maturers, those within the lower and upper bound limit of age were average maturers, whereas those above the upper bound limit of age were considered as delayed maturers.

In CS-3, much higher IGF-1 levels were found in delayed maturers (subjects above the upper bound limit of age). This finding can be useful in clinical orthodontic practice as in delayed type of maturers at CS-3 treatment should be started without delay. In delayed maturers at CS-3, mean height and weight also increased, which was possibly the explanation for increased IGF-1 levels in this group. The serum IGF-1 level was consistently on the lower hub in CS-5 in all three maturity groups, suggesting the fact that most patients attain their maximum growth by the time of CS-5. However, IGF-1 levels of early maturers were relatively higher than average and those of delayed type of maturers.

Another study [26] established mean serum IGF-1 levels for both sexes in all CVMI stages which were higher compared to those in our study; however, their method involved ELISA, a different technique to measure serum IGF-1 levels. The difference in mean serum IGF-1 levels may also be due to different study populations.

According to Ball et al. [11], on average, patients remain in CS-3 for 1.77 years and in CS-4 for 3.79 years. Often, we do not know at what time of the stage the cephalogram was taken, thus making it very difficult to assess the exact timing of growth spurt. The cervical vertebral maturation stages should be used along with other methods of biologic maturity assessment when considering both dentofacial orthopedic treatment and orthognathic surgery. The following combined classification may be used for selection of orthopedic and orthodontic treatment groups:

Group 1: cervical stages 3 and 4 with IGF-1 levels less than 310 ng/ml.

Group 2: cervical stages 3, 4, and 5 with IGF-1 levels more than 310 ng/ml.

Group 3: cervical stage 5 with IGF-1 levels less than 310 ng/ml.

Different clinical studies on the skeletal response of functional appliance have shown different treatment results; this may be due to the different designs of appliance, duration of treatment, patient's cooperation, type of malocclusion, and also on different maturational statuses as well as type of maturers. Hence, to reduce overall treatment timing, it would be better to follow new combined group criteria on the basis of cervical staging and quantitative assessment of IGF-1 levels. A patient showing CS-3 with serum IGF-1 levels less than 310 ng/ml can wait for IGF-1 level reevaluation to start treatment with functional appliance.

If a patient is at cervical stage 3, 4, or even 5 and has a serum IGF-1 level more than 310 ng/ml, treatment for functional jaw orthopedics can be chosen. If IGF-1 level is >310 ng/ml and cervical stage is 5, orthopedic treatment may be started without delay because very little time for growth is remaining. Since IGF-1 levels vary as per body type (athletic, aesthetic, and plethoric) and type of maturers (early, average, and late), longitudinal study with larger sample size consisting of similar body type and type of maturer might result in establishing a clinically useful reference range.

## Conclusions

Serum IGF-1 levels may be used as an additional tool for optimizing orthodontic treatment timing. Further studies with larger sample size are required to prove this.
